# Metropolis and kinetic Monte Carlo methods for studying protein interactions

**DOI:** 10.1007/s12551-026-01458-3

**Published:** 2026-09-16

**Authors:** Luciana Renata de Oliveira, Luiz Fernando de Camargo Rodrigues, Rosangela Itri

**Affiliations:** Institute of Physics, University of São Paulo, São Paulo, SP 05508-090 Brazil

**Keywords:** Monte Carlo simulations, Protein interactions, MMC, KMC

## Abstract

Protein–protein interactions are stochastic and play a central role in determining the structural and dynamic properties of biological systems. Computational approaches that account for the probabilistic nature of these interactions are therefore essential for understanding processes ranging from molecular self-assembly to biomolecular phase separation. Among these approaches, Monte Carlo (MC) methods have become important tools for investigating protein systems across multiple spatial and temporal scales. This review examines the application of MC simulations to protein–protein interactions, with particular emphasis on Metropolis Monte Carlo (MMC) and kinetic Monte Carlo (KMC) methods as complementary approaches for investigating equilibrium properties and dynamic molecular processes. Applications ranging from receptor organization and protein aggregation to biomolecular condensation and liquid–liquid phase separation are discussed together with the evolution of coarse-grained models, from isotropic particles to anisotropic patchy particles and polymer representations that capture directional, multivalent, and sequence-dependent interactions. Finally, the integration of MC simulations with experimental techniques is reviewed through applications involving Small Angle X-ray and Neutron Scattering (SAXS/SANS), single-molecule tracking, and fluorescence-based methods, highlighting how experimentally accessible observables can be directly compared with simulation results to characterize protein interactions and estimate molecular parameters.

## Introduction

Biological systems are stochastic due to the random nature of molecular interactions, diffusion processes, and biochemical reactions. This stochasticity is particularly relevant in protein interaction studies, where binding events, conformational changes, and reaction dynamics are often influenced by probabilistic processes (Amjad & Sokouti 2025; Basak et al. 2019; Bressloff 2021). In this way, computational models that can incorporate randomness are important for describing these systems. Among the available stochastic approaches, Monte Carlo (MC) simulation has become an important tool for understanding the behavior of complex physical, chemical, and biological systems (Meng et al. 2004; Székely & Burrage 2014; Ullah & Wolkenhauer 2010). Broadly, MC encompasses a range of iterative computational techniques that employ random sampling to explore system behavior and estimate quantities that may be difficult or impossible to obtain using deterministic algorithms (Liu 2004; Rubinstein & Kroese 2016; Shreider 1966) (Liu 2004; Rubinstein & Kroese 2016; Shreider 1966).

The development of MC methods began during World War II, when Stanislaw Ulam and John von Neumann proposed MC simulations to address mathematical and physical problems that were computationally impossible to solve using conventional analytical approaches (Metropolis & Ulam 1949; Von Neumann 1963). The term “Monte Carlo” was later coined by Nicholas Metropolis in reference to the district of Monaco, highlighting the fundamental role of randomness and probabilistic sampling in the method.

**Fig. 1:**
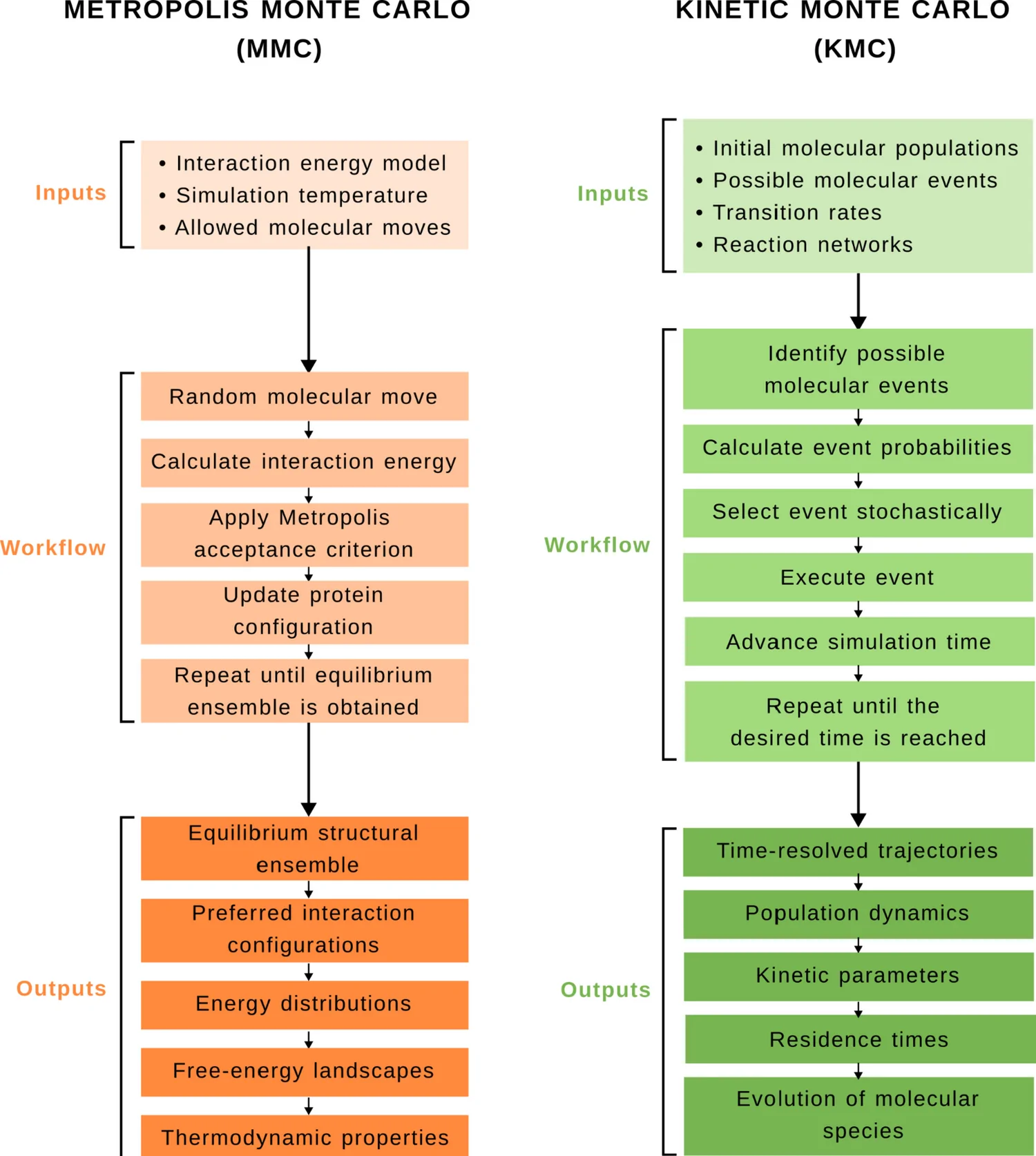
Schematic comparison of Metropolis Monte Carlo (MMC) and kinetic Monte Carlo (KMC) methods, including their inputs, workflows, and outputs

Among the broad range of Monte Carlo techniques, this review focuses specifically on two widely used approaches, Metropolis Monte Carlo (MMC) and kinetic Monte Carlo (KMC), which differ primarily in how they describe system evolution and in the role assigned to time during the simulation (Fig. 1). MMC, originally introduced by Metropolis et al. (1953), is based on the evaluation of energy changes between system configurations and the application of an acceptance criterion that satisfies detailed balance, enabling efficient sampling of equilibrium distributions. In contrast, KMC is explicitly designed to describe the time evolution of stochastic systems via discrete events governed by transition rates. Thus, while MMC and KMC both rely on stochastic sampling, they address fundamentally different modeling objectives: MMC primarily characterizes equilibrium ensembles, whereas KMC can describe dynamic processes when a physically meaningful set of transition rates is available.

In a typical MMC simulation (Fig. 1), the inputs consist of an interaction energy model, the simulation temperature, and a set of allowed moves that define how the system can explore configurational space. Starting from an initial system configuration, a random move is proposed, such as, in the case of proteins, a conformational change, displacement, rotation, or modification of intermolecular contacts. The interaction energy of the new configuration is then calculated and compared with that of the previous state.

The acceptance or rejection of the proposed move is determined according to the Metropolis criterion, in which energetically favorable moves are always accepted. In contrast, unfavorable moves are accepted with a probability given by the Boltzmann factor, $P=exp(-\Delta E/{k}_{B}T)$, where ΔE is the energy difference between configurations, ${k}_{B}$ is the Boltzmann constant, and *T* is the simulation temperature. If the move is accepted, the system configuration is updated, otherwise, the previous configuration is retained. This procedure is repeated iteratively until an equilibrium ensemble is obtained, ensuring that sampled configurations follow the Boltzmann distribution.

While MMC focuses on equilibrium sampling, KMC methods were developed to describe the stochastic evolution of systems governed by discrete events occurring at known transition rates. The foundations of the method can be traced to the work of Young and Elcock (1966), who introduced a stochastic framework for simulating vacancy migration in alloys. Later, Bortz et al. (1975) developed the n-fold way algorithm, establishing the basis of modern event-driven KMC simulations. Subsequent advances, including the Gillespie algorithm for chemical reaction systems (Gillespie 1977), extended the applicability of KMC methods to biological and biochemical processes.

Unlike MMC, KMC operates as a dynamic, rate-driven framework in which system evolution is determined by stochastic transitions between discrete states (Andersen et al. 2019). Rather than sampling configurational space to obtain equilibrium properties, KMC models biological processes as networks of events governed by experimentally measured or estimated transition rates (Resat et al. 2009).

In practice, a KMC simulation (Fig. 1) begins by defining an initial molecular population, a set of possible molecular events, their associated transition rates, and the underlying reaction network. At each simulation step, all events that can occur from the current system state are identified, and their probabilities are calculated from the corresponding rate constants. An event is then selected stochastically according to these probabilities, typically using Gillespie-type algorithms or equivalent rejection-free approaches (Vestergaard & Génois 2015). Once the selected event is executed, the molecular populations and system state are updated.

The simulation clock is subsequently advanced by a time increment sampled from an exponential distribution with a mean equal to the total event rate, consistent with the Poisson statistics governing independent stochastic processes (Pettigrew & Resat 2007). This procedure is repeated iteratively until the desired simulation time is reached, generating a statistically representative trajectory of the system evolution. Because KMC explicitly incorporates transition rates and time progression, it provides direct access to time-resolved trajectories, population dynamics, kinetic parameters, residence times, and the evolution of molecular species.

Since their development, Monte Carlo methods have been successfully adapted to investigate complex biological systems across multiple spatial and temporal scales. The complementary strengths of MMC and KMC approaches enable the characterization of both equilibrium properties and dynamic processes, making them valuable tools for studying protein conformational changes, protein–protein interactions, aggregation, self-assembly, and biomolecular reaction networks (Gillespie 1977; Grassmann et al. 2024; Liang & Qian 2010; Loskot et al. 2019; Zhou & Wang 2026). These applications should be considered with other computational approaches commonly used to investigate protein interactions, each of which offers distinct advantages and limitations.

Molecular dynamics (MD) and Brownian dynamics (BD) are also widely used to study protein interactions, but address different aspects of biomolecular behavior and have different applicability. MD provides detailed information on conformational dynamics and interaction mechanisms by explicitly propagating molecular coordinates in time, but adequate sampling of slow transitions, rare events, or complex association processes can require long and computationally demanding simulations (Badar et al. 2022). BD is particularly suited to studying diffusional association and encounter processes, often using simplified molecular and solvent representations to access longer spatial and temporal scales (Muñiz‐Chicharro et al. 2023). In contrast, MC focuses on configurational sampling rather than physical time evolution, making it particularly useful for characterizing equilibrium ensembles and interaction configurations. Thus, MC complements rather than replaces MD and BD, with its main advantages arising when extensive configurational sampling is required.

Together, the complementary features of MMC and KMC make Monte Carlo methods valuable for studying a wide range of biophysical problems. Their flexibility and computational efficiency have also facilitated their integration with experimental data, providing quantitative insights into molecular mechanisms that are often difficult to access directly. In this review, particular emphasis is given to applications in protein aggregation, oligomerization, signaling, and interaction dynamics, as well as recent developments in the use of MC to study liquid–liquid phase separation, particle-based models, and their integration with experimental approaches.

## Applications of Monte Carlo methods to protein organization and assembly

Protein–protein interactions occur across multiple spatial and temporal scales, ranging from local conformational rearrangements to oligomerization, large-scale aggregation, and intracellular organization (Keskin et al. 2005). MC methods have been widely applied to investigate these processes by combining coarse-grained models with stochastic sampling schemes. Applications have evolved from receptor organization and signaling to protein aggregation, oligomerization, biomolecular reaction networks, and, more recently, liquid–liquid phase separation (LLPS), driving the development of increasingly sophisticated models for multivalent and anisotropic protein interactions. Representative examples of these applications are discussed below.

Early applications of MMC focused on receptor organization and signaling, using equilibrium sampling to investigate how molecular interactions drive clustering and spatial organization at the cell membrane. Guo and Levine (2000) used a lattice-based MMC model to sample receptor distributions on cell membranes and demonstrated that weak receptor–receptor interactions are sufficient to drive stable clustering and large-scale spatial organization.

Extending this approach, Weikl and Lipowsky (2004) applied MMC to a statistical-mechanical model of T-cell adhesion, showing that thermodynamic interactions can drive spontaneous formation and coalescence of receptor microdomains into immunological synapse patterns. Lee et al. (2003) further employed MC simulations to investigate receptor redistribution within the immunological synapse, revealing how spatial segregation regulates signaling and receptor turnover.

Similarly, Qi et al. (2001) used stochastic MC simulations to model receptor–ligand interactions at the cell–cell interface and demonstrated that synaptic organization emerges through self-assembly governed by the intrinsic physicochemical properties of the system.

Beyond receptor organization, MMC simulations have been widely applied to investigate protein aggregation, providing molecular insight into the structural mechanisms underlying oligomerization, nucleation, and fibril formation. Dey and Biswas (2023) sampled peptide conformations and intermolecular arrangements of amyloid-β42, revealing that hydrophobic interactions and cooperative structural rearrangements promote the formation of stable oligomeric and β-sheet-rich aggregates. Likewise, Okumura et al. (2026) developed a lattice-based MMC model in which proteins transition between monomeric, intermediate, and fibrillar states, showing how transition probabilities control nucleation and fibril growth kinetics.

Extending these studies to dynamic processes, KMC methods have enabled the investigation of protein aggregation kinetics, oligomerization pathways, and stochastic biochemical reactions. Li et al. (2008) employed MC simulations to investigate the early stages of oligomerization of the amyloidogenic tau fragment AcPHF6, revealing that aggregation proceeds through transient metastable oligomers that undergo structural reorganization before forming stable β-sheet-rich assemblies. Tiwari and van der Schoot (2016) applied KMC simulations to nucleated self-assembly processes and demonstrated that lag times in protein aggregation are broadly distributed due to stochastic fluctuations, with variability strongly dependent on system size and aggregation pathways.

Liu and Kang (2020) and Ding et al. (2024) developed stochastic master-equation frameworks to investigate amyloid oligomer formation, showing that oligomer populations are highly sensitive to monomer availability, protein misfolding, and secondary nucleation processes, which act as major drivers of aggregate amplification. Bodner et al. (2023) used MC simulations to model diffusion and photobleaching in membrane protein oligomerization experiments, identifying key sources of measurement bias and establishing optimized conditions for accurate oligomer detection. More recently, Peter et al. (2026) employed MC simulations to investigate the spatial propagation of Aβ42 aggregation, revealing that soluble oligomers generated through secondary nucleation act as mobile carriers that accelerate the spread of amyloid formation across space. Overall, these studies demonstrate that MC models and efficient sampling can capture complex protein interaction landscapes and large-scale processes. However, their predictive accuracy and transferability may be limited by reduced molecular detail and poorly constrained interaction potentials or transition rates.

Beyond aggregation, multivalent protein interactions can also drive liquid–liquid phase separation (LLPS), producing dynamic biomolecular condensates driven by weak and transient interactions that play essential roles in cellular organization. Since its experimental observation in vivo (Brangwynne et al. 2009), LLPS has become an important area of investigation for both in vitro and in silico experiments, including with MC methods. Along with these findings, more complex coarse-grained representations of proteins have been developed to capture the anisotropic and multivalent nature of this phenomenon, with the employment of both colloidal and polymeric models (Ranganathan & Shakhnovich 2020; Stradner & Schurtenberger 2020).

**Fig. 2:**
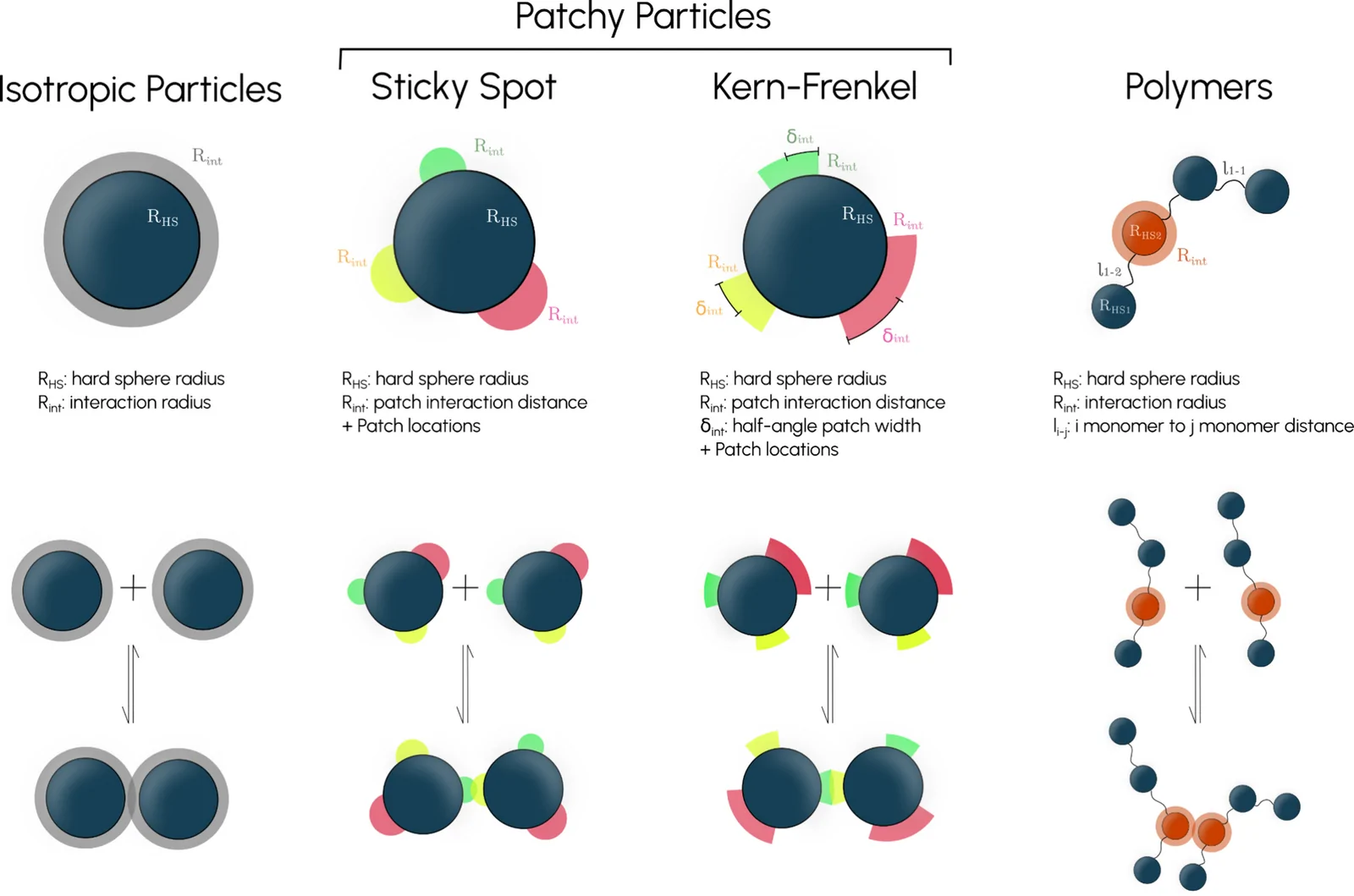
Representative coarse-grained models used in MC simulations of protein–protein interactions. These representations can be combined with Metropolis or kinetic Monte Carlo schemes to stochastically sample configurational space or simulate the temporal evolution of protein assemblies. Isotropic particles interact without orientation dependence, whereas patchy particles introduce directional interactions that depend on both particle distance and the relative positions of their binding sites. Polymer models explicitly account for chain connectivity and multivalent interactions through interacting subunits. In the example shown, the orange subunits represent “stickers” and the blue subunits represent “spacers,” according to the stickers-and-spacers model

Figure 2 summarizes the most commonly used coarse-grained representations currently employed in MC simulations of protein–protein interactions. Regardless of the level of coarse-graining, these models are coupled to MC sampling schemes that stochastically generate new configurations, enabling either equilibrium sampling or the simulation of time-dependent molecular processes. Model complexity ranges from isotropically interacting colloids, in which interactions depend only on particle distance, to patchy particle models that introduce directional binding and polymer-based representations that explicitly account for chain connectivity and multivalent interactions per chain. In patchy particle models, interaction sites may be represented as discrete attractive patches (“sticky spots”) attached to the particle surface (Rovigatti et al. 2018; Teixeira & Tavares 2017) or as angular interaction regions as defined in the Kern–Frenkel framework (Kern & Frenkel 2003), thereby introducing anisotropic interactions that occur only when particles adopt appropriate relative orientations. Polymer models explicitly account for each unit of the polymeric chain, employing a fundamentally different strategy to describe the protein shape.

It is important to observe that, since proteins are polymers, employing the colloidal approach, approximating these macromolecules as spheres, brings important limitations. This is especially relevant regarding many occurrences of LLPS, considering the important role of flexibility in intrinsically disordered regions for transient interactions to take place and induce phase separation (Brangwynne et al. 2015; Pappu et al. 2023). Despite these limitations, advances in the approaches described above and the lower computational cost of most colloidal models make them valuable for studying systems in which these approximations are justified. When the target proteins can be parametrized in terms of colloids, this approach is especially useful for simulating many particles for longer timescales and observing the role of specific changes in the medium (such as pH and temperature) or in specific particle features (size, binding energies etc.) in the overall system behavior.

The models described above have been used both for molecular dynamics (MD) and MC simulations to investigate LLPS and protein–protein interactions in general. Simulations with interactions being mediated by different isotropic particles in the so-called “floating bonds” model (Zaccarelli et al. 2007) have been used to study the association of globular proteins with trivalent salts (Bleibel et al. 2018). Ranganathan and Shakhnovich (2020) employed a KMC model with specific bond formation between diffusing particles on a lattice to construct phase diagrams and showed how particle density, bonding rate, and valency govern phase separation behavior. The “stickers-and-spacers” framework subsequently provided a conceptual basis for describing LLPS in terms of the balance between intra- and intermolecular interactions among associative motifs (Brangwynne et al. 2015; Pappu et al. 2023).

Polymer-based models were also employed to better represent intrinsically disordered proteins and multivalent interactions, and Choi et al. (2019) developed LASSI, building on the “stickers-and-spacers” framework: a lattice-based MC platform that simulates polymer dynamics through translation, rotation, and slithering-snake moves, enabling the characterization of gelation and phase separation into condensed protein-rich phases. Using related lattice-polymer approaches, Rana et al. (2021) applied grand canonical MC simulations to investigate the effects of sequence length, composition, and charge distribution on phase behavior, distinguishing LLPS from cluster-forming aggregation.

More recently, patchy particle models have been extended to incorporate a degree of structural flexibility. Taskina et al. (2025) introduced dynamic patchy particle models that account for fluctuating binding-site positions and interaction ranges, improving the representation of structural flexibility in MD simulations of biomolecular condensates, suggesting an alternative modeling strategy that may complement future Monte Carlo simulations.

Collectively, these studies illustrate the evolution of MC models from simple lattice descriptions to increasingly realistic coarse-grained representations capable of capturing the multivalency and anisotropy underlying protein–protein interactions and biomolecular condensation. However, increasing model complexity does not necessarily translate into greater predictive accuracy, as phase behavior can remain highly sensitive to the choice of coarse-grained representation, interaction potentials, and model parameters. Closer integration with experimental data is therefore essential to constrain and validate these models.

## Combining Monte Carlo simulations and experimental approaches for protein–protein interaction studies

**Fig. 3:**
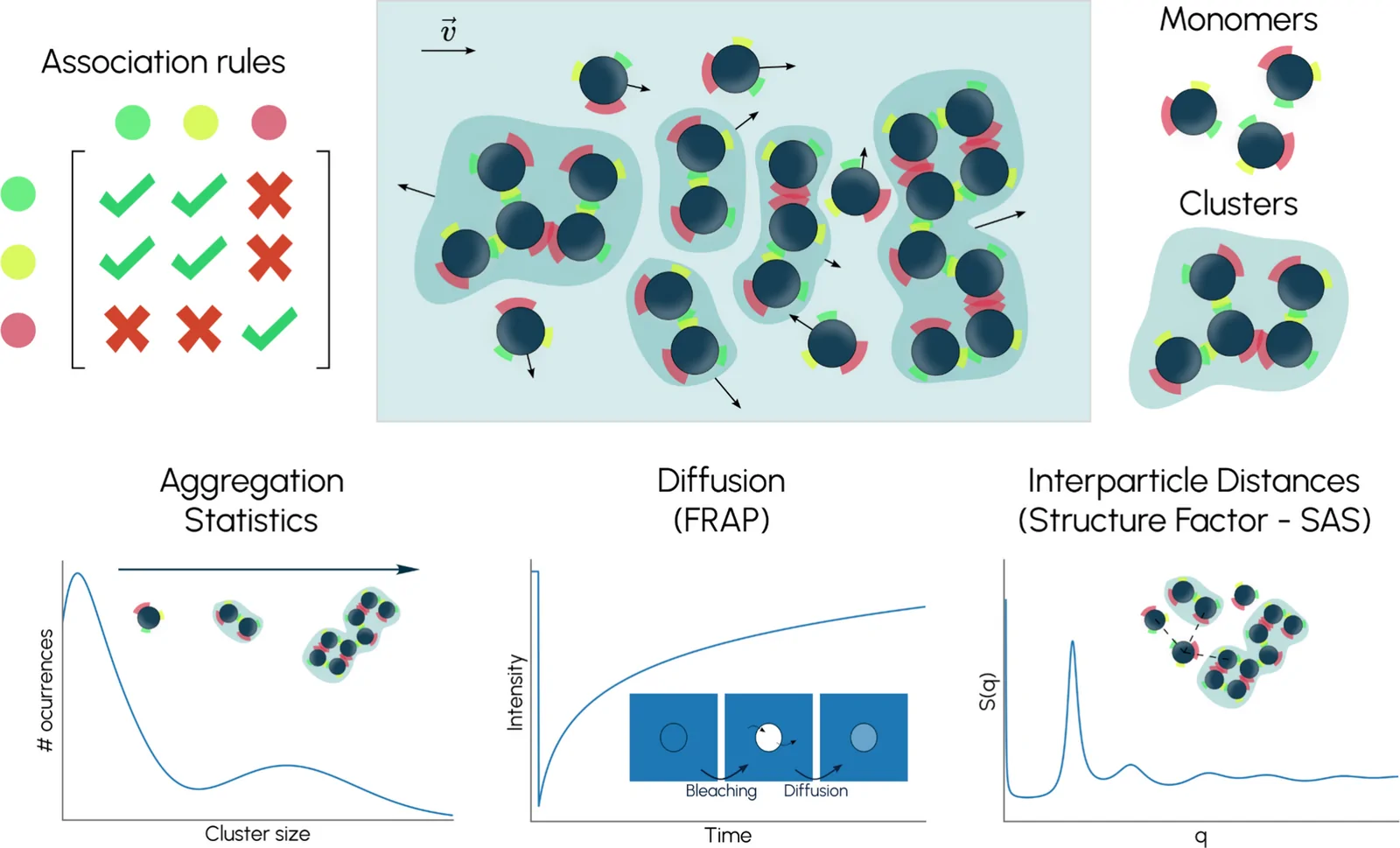
From particle-based simulations to experimentally accessible observables. Patchy particles diffuse and associate according to predefined interaction rules and energies, forming clusters of different sizes. Cluster-size distributions can be obtained directly from the simulated assemblies and used to characterize aggregation behavior. Particle trajectories can also be analyzed to reproduce fluorescence recovery after photobleaching (FRAP) measurements by monitoring molecular exchange within a selected region. Furthermore, interparticle distance distributions enable the calculation of the structure factor, S(q), which can be directly compared with Small-Angle X-ray and Neutron Scattering (SAXS/SANS) experiments. These observables provide complementary information on the structural organization and dynamics of protein assemblies

The increasing realism and applicability of particle-based MC models has enabled their integration with experimental techniques, allowing simulations and experiments to be connected through common observables. Rather than providing only qualitative descriptions of protein assemblies, MC simulations can generate experimentally accessible quantities that are used to assess computational models, constrain molecular parameters, and interpret the mechanisms underlying protein–protein interactions. As illustrated in Fig. 3, the same simulation framework can produce observables such as cluster-size distributions, fluorescence recovery curves, and scattering structure factors, providing complementary information on both the structural organization and dynamic behavior of protein assemblies.

However, the agreement between simulated and experimental observables does not imply that the underlying model or parameters are uniquely determined. The inference of parameters may be limited when different combinations of interaction potentials or molecular parameters lead to similar experimental observables (Mitra & Hlavacek 2019). In addition, uncertainties in experimental measurements, model assumptions, and parameter estimation can propagate to predicted molecular properties (Hines et al. 2014). Thus, combining multiple independent experimental observables and explicit assessment of parameter uncertainty are important aspects of robust model validation and differentiating well-constrained molecular mechanisms from alternative explanations (Vlcek et al. 2017).

An approach that allows for bypassing potential choice issues is the Reverse Monte Carlo (RMC) method: if there are experimental data for the system and forward models to calculate these quantities from a model (for example, a spatial configuration of particles), instead of minimizing an energy, the algorithm seeks to minimize some goodness of fit quantity, such as χ^2^, of the calculated model compared to the data (McGreevy 2001; McGreevy & Pusztai 1988). The process is iteratively repeated until no further improvement is noticed on the fit. Therefore, it is a method of solving inverse problems. Although issues such as avoiding local minima, relative abundance of free parameters and limitations on forward modelling may be present (and mitigated in various ways), different problems have been tackled with Reverse Monte Carlo, especially on structural modelling using total scattering and absorption techniques in condensed matter, by means of the pair-distribution function (g(r)) and the structure factor (S(q)) (Bousige et al. 2015; Hara et al. 2025; Winterer & Geiß 2023). An illustrative example in a correlated area is shown at the end of the “Scattering Techniques (SAXS/SANS)”.

The following sections illustrate how these simulation-derived observables are integrated with different experimental techniques to characterize complementary aspects of protein–protein interactions, including structural organization, molecular diffusion, and interaction kinetics.

### Scattering techniques (SAXS/SANS)

Among the observables illustrated in Fig. 3, the structure factor (S(q)) provides a direct connection between MC simulations and scattering experiments. Since many questions concerning protein–protein interactions are related to the structures that emerge from these interactions, techniques that probe these features are highly desirable. Small Angle X-Ray/Neutron Scattering (SAXS/SANS) is widely used in structural biology and soft matter for studying questions related to protein structure (Petoukhov & Svergun 2013), dynamics of oligomerization (Asor et al. 2020), flexibility (Bernadó et al. 2007; Tria et al. 2015), and the formation of larger molecular assemblies (Asor et al. 2020; Shemesh et al. 2021). The scattering measured in SAXS/SANS is directly related to the ensemble-averaged spatial distribution of scattering density elements, corresponding to electron density in SAXS and to neutron scattering length density in SANS. Therefore, SAXS/SANS brings experimental information on interparticle distances, which can be measured experimentally and also calculated analytically (when possible, from a wide range of analytical models), or obtained directly from simulations, enabling direct comparison with experimental results.

Tanouye et al. (2023) employed a combination of SAXS measurements and MC simulations to characterize lysozyme–lysozyme interactions across different solution conditions, comparing simulation results with analytical models to assess the role of net charge-driven interactions between lysozyme molecules. The simulation box was filled with spherical proteins and ions, using a potential with a hard-sphere term and a screened Coulomb term for the interaction potential. The comparison between simulated and experimental scattering profiles revealed limitations of analytical closure relations commonly used to extract interaction potentials. Another study for lysozyme using the same experimental data was done by Qin and Zhou, but this time, the objective was to speed up simulations for all-atom dense protein simulations (Qin & Zhou 2025). Following a previous study on the calculation of structure factors (Qin & Zhou 2024), using a potential with hard-sphere, Lennard–Jones, and Debye-Hückel terms, the group performed MC simulations of the system with precomputed protein pair interaction energies for different distances and relative orientations on a grid. During the simulations, the particles were moved according to the Metropolis criterion, and the potential change was calculated by using the corresponding value from the precomputed lookup table instead of recalculating from scratch the pair potential contribution, speeding up the calculations and allowing faster simulations.

Studies for patchy particles have also been performed, probing the emerging structure factor from simulations, employing models such as the floating bonds model (Bleibel et al. 2018) or a rigid multi-bead model for studying antibody aggregation in high concentrations, a problem of special pharmaceutical interest (Skar-Gislinge et al. 2019, 2023). In these studies, Skar-Gislinge et al*.* have defined patches based on the properties of the calculated electrostatic potential, observing two positive and one negative regions on opposite sides of the Y-shaped antibody, approximating its shape by a combination of beads or effective hard spheres. Combining Static and Dynamic Light Scattering, Microrheology, and SAXS with MC simulations of patchy particles, Skar-Gislinge et al. studied the self-assembly behavior of antibodies in high concentrations, also developing an analytical approach for calculating the structure factor analytically from polymer theory for this type of system.

Beyond the scope of protein–protein interactions, the topic of protein structure (through the scattering form factor, P(q)) determination at low resolution brings interesting elements: the very well-established ATSAS suite contains diverse tools for analyzing protein structure with SAXS/SANS (Franke et al. 2025). Some of such pieces of software are DAMMIN/DAMMIF and GASBOR, which employ dummy atoms (or dummy residues) as volume elements for describing a protein envelope following an experimental scattering curve (Franke & Svergun 2009; Svergun 1999; Svergun et al. 2001). The programs perform simulated annealing similarly to the RMC methods mentioned above: the protein structure is represented by volume elements with specific constant scattering length densities. In an iterative process, the protein envelope configuration (through its volume elements) is changed, the form factor of this new configuration is calculated and, subsequently, χ^2^, comparing the model to the data. Using the Metropolis acceptance criterion with χ^2^ as the energy to avoid shallow local minima, the procedure persists for a certain number of steps. Then, following the simulated annealing procedure, the temperature is reduced periodically at a predefined rate until a final structure is obtained. Normally, due to multiple solutions being achievable through this method, multiple structures are calculated and an average structure is obtained (with the option of applying clusterization for classifying very different resulting structures). This average is used as the starting point for a last minimization, which results in a refined structure. This approach makes direct use of experimental data for modeling the protein, guiding the Monte Carlo simulation as a potential would in MMC.

In the field of soft matter, nanoparticles and polymers, RMC has been used by some groups to understand aggregation phenomena and the spatial distribution of particles. For this, SAXS/SANS data are especially valuable as a reference for guiding the simulations, and a number of different groups not only fit their data by simulating each particle or cluster of particles using RMC (Kruteva et al. 2022; Musino et al. 2018), but also perform MC simulations as verification strategies for analytical models, also by cross-checking parameters from the model simulated in MC (Pauw et al. 2013; Pedersen et al. 2026).

### Single-molecule tracking and diffusion measurements

While scattering experiments primarily probe structural organization, single-molecule tracking provides direct information on molecular mobility and diffusion. Single-molecule tracking techniques are used to determine protein mobility, receptor dynamics, and membrane-associated interactions (Nguyen et al. 2023; Simon et al. 2024). MC methods have been applied to interpret these measurements and extract kinetic parameters that are difficult to obtain directly from experimental observations. de Oliveira and Jaqaman (2019) combined KMC simulations with single-molecule imaging data to investigate molecular interaction kinetics on cell membranes. The simulations modeled molecular diffusion and oligomerization dynamics, generating trajectories that were compared with experimental single-particle tracking and oligomer distribution data. Similarly, Weimann et al. (2013) combined MC simulations with single-particle tracking experiments to investigate the diffusion dynamics of lipopolysaccharide receptors in live macrophages. The simulations generated synthetic trajectories with known diffusion properties that were compared with experimental tracking data and used to evaluate different mobility analysis methods. Chong et al. (2022) combined KMC simulations with single-particle tracking measurements of quantum dot-labeled cytochromes to investigate long-range electron transport in bacterial membranes. The simulations incorporated experimentally determined diffusion coefficients and modeled electron hopping between neighboring redox-active proteins.

### Fluorescence-based techniques

Complementing structural and diffusion measurements, fluorescence-based techniques provide quantitative information on protein turnover, molecular interactions, and conformational dynamics. MC methods have also been applied to the interpretation of fluorescence-based experiments. de Oliveira et al. (2025) combined KMC simulations based on the Gillespie algorithm with Fluorescence Recovery After Photobleaching (FRAP) and Fluorescence Loss After Photoactivation (FLAP) measurements of focal adhesion proteins to investigate protein turnover and interactions. The simulations modeled stochastic protein interactions and turnover, while experimental fluorescence data were used to estimate kinetic parameters. Hibbard et al. (2021) also combined MC simulations with FRAP experiments to investigate the dynamics of intraflagellar transport proteins at basal bodies. The simulations modeled protein diffusion and transient capture at binding sites, while experimental recovery curves were used to characterize protein turnover. Thompson et al. (2020) combined single-molecule FRET measurements with kMC simulations to investigate E-cadherin clustering at cell membranes. The simulations modeled molecular association and dissociation processes under different interaction scenarios, while experimental data provided cluster size distributions and interaction dynamics.

In a complementary approach, Hoefling et al. (2011) combined single-molecule FRET experiments, MD and MC simulations to investigate the structural heterogeneity of polyproline peptides. MC simulations sampled the positions and orientations of fluorescent probes, while molecular dynamics provided conformational ensembles that were compared with experimental FRET measurements.

## Conclusions

MC methods have become important tools for studying protein–protein interactions across multiple spatial and temporal scales. MMC and KMC provide complementary frameworks for investigating equilibrium properties and dynamic molecular processes. As discussed throughout this review, their applications have expanded from receptor organization and protein aggregation to biomolecular condensation, self-assembly, and interaction kinetics.

This evolution has been accompanied by the development of increasingly realistic coarse-grained models. Simpler lattice representations and isotropic particles have progressed to patchy particle and polymer models that capture anisotropy, multivalency, and sequence-dependent interactions. These advances have amplified the range and realism of biological systems that can be investigated while maintaining computational efficiency. However, increasing model complexity also introduces challenges related to parameterization, transferability, and validation. Simplifications inherent to coarse-grained representations may affect the description of molecular interactions, and predictions can depend strongly on the choice of interaction potentials, model parameters, and sampling strategies. Thus, improving the balance between computational efficiency and molecular detail remains an important challenge.

An important advance has been the integration of MC simulations with experimental techniques. Simulations can generate observables that are directly comparable with experiments, including scattering profiles, fluorescence recovery curves, diffusion trajectories, and cluster size distributions. This integration allows the estimation of interaction energies, kinetic rate constants, diffusion coefficients, and other molecular parameters that are difficult to obtain experimentally. Nevertheless, further efforts are needed to systematically quantify uncertainties associated with model assumptions, parameter estimates, and sampling. In particular, establishing robust connections between simulated trajectories or Monte Carlo steps and experimentally measurable timescales remains challenging, especially for systems in which transition rates are not directly known.

Reverse Monte Carlo approaches, even though avoiding working directly with potentials, work directly with experimental data as constraints guiding the simulations and may aid in bringing more ideas to the table with their recent advances, both computational and theoretical (Agrahari & Chatterjee 2021; Cuillier et al. 2024). Advances in how molecular dynamics simulations handle experimental constraints, employing them as a bias term in the potential for energy calculations to guide protein conformation sampling (Boomsma et al. 2014; Chatzimagas & Hub 2023; Hsu et al. 2021; Zhao & Shukla 2018), may also bring alternatives for how to adapt MC methods for protein–protein interaction questions.

Future developments will likely benefit from improved coarse-grained models and multiscale approaches that combine different levels of molecular resolution to bridge atomistic, mesoscale, and cellular descriptions. Such approaches could provide a more realistic representation of protein interactions while retaining the computational efficiency required to explore large systems and long timescales.

## Data Availability

No datasets were generated or analysed during the current study.

## References

[CR1] Agrahari G, Chatterjee A (2021) Thermodynamic calculations using reverse Monte Carlo. Phys Rev E 104(4–1):044129. 10.1103/PhysRevE.104.044129 34781448

[CR2] Amjad E, Sokouti B (2025) Kinetics and dynamics of biological systems. Systems biology and in-depth applications for unlocking diseases. Elsevier, pp 59–67. 10.1016/B978-0-443-22326-6.00006-7

[CR3] Andersen M, Panosetti C, Reuter K (2019) A practical guide to surface kinetic Monte Carlo simulations. Front Chem 7:202. 10.3389/fchem.2019.00202 PMC6465329 31024891

[CR4] Asor R, Schlicksup CJ, Zhao Z, Zlotnick A, Raviv U (2020) Rapidly forming early intermediate structures dictate the pathway of capsid assembly. J Am Chem Soc 142(17):7868–7882. 10.1021/jacs.0c01092 PMC7242811 32233479

[CR5] Badar MS, Shamsi S, Ahmed J, Alam M (2022) Molecular dynamics simulations: concept, methods, and applications. In: Rezaei N (ed) Transdisciplinarity, vol Vol. 5. Springer International Publishing, Cham, pp 131–151. 10.1007/978-3-030-94651-7_7

[CR6] Basak S, Sengupta S, Chattopadhyay K (2019) Understanding biochemical processes in the presence of sub-diffusive behavior of biomolecules in solution and living cells. Biophys Rev 11(6):851–872. 10.1007/s12551-019-00580-9 PMC6957588 31444739

[CR7] Bernadó P, Mylonas E, Petoukhov MV, Blackledge M, Svergun DI (2007) Structural characterization of flexible proteins using small-angle X-ray scattering. J Am Chem Soc 129(17):5656–5664. 10.1021/ja069124n 17411046

[CR8] Bleibel J, Habiger M, Lütje M, Hirschmann F, Roosen-Runge F et al (2018) Two time scales for self and collective diffusion near the critical point in a simple patchy model for proteins with floating bonds. Soft Matter 14(39):8006–8016. 10.1039/c8sm00599k 30187060

[CR9] Bodner C, Kiesenhofer D, Schütz GJ, Brameshuber M (2023) Monte Carlo simulations for the evaluation of oligomerization data in TOCCSL experiments. Biophys J 122(11):2367–2380. 10.1016/j.bpj.2023.04.021 PMC10257147 37088991

[CR10] Boomsma W, Ferkinghoff-Borg J, Lindorff-Larsen K (2014) Combining experiments and simulations using the maximum entropy principle. PLoS Comput Biol 10(2):e1003406. 10.1371/journal.pcbi.1003406 PMC3930489 24586124

[CR11] Bortz AB, Kalos MH, Lebowitz JL (1975) A new algorithm for Monte Carlo simulation of Ising spin systems. J Comput Phys 17(1):10–18. 10.1016/0021-9991(75)90060-1

[CR12] Bousige C, Boţan A, Ulm F-J, Pellenq R-M, Coasne B (2015) Optimized molecular reconstruction procedure combining hybrid reverse Monte Carlo and molecular dynamics. J Chem Phys 142(11):114112. 10.1063/1.4914921 25796236

[CR13] Brangwynne CP, Eckmann CR, Courson DS, Rybarska A, Hoege C et al (2009) Germline P granules are liquid droplets that localize by controlled dissolution/condensation. Science 324(5935):1729–1732. 10.1126/science.1172046 19460965

[CR14] Brangwynne CP, Tompa P, Pappu RV (2015) Polymer physics of intracellular phase transitions. Nat Phys 11(11):899–904. 10.1038/nphys3532

[CR15] Bressloff PC (2021) Stochastic processes in cell biology: volume I, Interdiscip Appl Math, vol 41. Springer International Publishing, Cham. 10.1007/978-3-030-72515-0

[CR16] Chatzimagas L, Hub JS (2023) Structure and ensemble refinement against SAXS data: combining MD simulations with Bayesian inference or with the maximum entropy principle. Methods Enzymol 678:23–54. 10.1016/bs.mie.2022.09.014 36641209

[CR17] Choi J-M, Dar F, Pappu RV (2019) LASSI: a lattice model for simulating phase transitions of multivalent proteins. PLoS Comput Biol 15(10):e1007028. 10.1371/journal.pcbi.1007028 PMC6822780 31634364

[CR18] Chong GW, Pirbadian S, Zhao Y, Zacharoff LA, Pinaud F, El-Naggar MY (2022) Single molecule tracking of bacterial cell surface cytochromes reveals dynamics that impact long-distance electron transport. Proc Natl Acad Sci U S A 119(19):e2119964119. 10.1073/pnas.2119964119 PMC9171617 35503913

[CR19] Cuillier P, Tucker MG, Zhang Y (2024) Integrating machine learning interatomic potentials with hybrid reverse Monte Carlo structure refinements in RMCProfile. J Appl Crystallogr 57(Pt 6):1780–1788. 10.1107/S1600576724009282 PMC11611278 39628890

[CR20] Dey P, Biswas P (2023) Exploring the aggregation of amyloid-β 42 through Monte Carlo simulations. Biophys Chem 297:107011. 10.1016/j.bpc.2023.107011 37037120

[CR21] de Oliveira LR, Fernandes MG, Patane JSL, Schwartz J-M, Krieger JE et al (2025) Exploring focal adhesion data: dynamic parameter extraction from FRAP and FLAP experiments using chemical master equation. Front Mol Biosci 12:1587608. 10.3389/fmolb.2025.1587608 PMC12088951 40396124

[CR22] de Oliveira LR, Jaqaman K (2019) FISIK: framework for the inference of in situ interaction kinetics from single-molecule imaging data. Biophys J 117(6):1012–1028. 10.1016/j.bpj.2019.07.050 PMC6818184 31443908

[CR23] Ding Y, Cai L, Kang Y (2024) Moment dynamics of oligomer formation in protein amyloid aggregation with secondary nucleation. Adv Contin Discret Model 2024(1):25. 10.1186/s13662-024-03819-2

[CR24] Franke D, Gräwert T, Svergun DI (2025) New features in ATSAS-4, a program suite for small-angle scattering data analysis. J Appl Crystallogr 58(Pt 3):1027–1033. 10.1107/S1600576725002481 PMC12135997 40475945

[CR25] Franke D, Svergun DI (2009) DAMMIF, a program for rapid ab-initio shape determination in small-angle scattering. J Appl Crystallogr 42(2):342–346. 10.1107/S0021889809000338 PMC5023043 27630371

[CR26] Gillespie DT (1977) Exact stochastic simulation of coupled chemical reactions. J Phys Chem 81(25):2340–2361. 10.1021/j100540a008

[CR27] Grassmann G, Miotto M, Desantis F, Di Rienzo L, Tartaglia GG et al (2024) Computational approaches to predict protein-protein interactions in crowded cellular environments. Chem Rev 124(7):3932–3977. 10.1021/acs.chemrev.3c00550 PMC11009965 38535831

[CR28] Guo C, Levine H (2000) A statistical mechanics model for receptor clustering. J Biol Phys 26(3):219–234. 10.1023/a:1010313529687 PMC3456755 23345723

[CR29] Hara N, Iesari F, Okajima T, Di Cicco A (2025) Local structure of solid and liquid gold probed by reverse Monte Carlo analysis of X-ray absorption data. J Synchrotron Radiat 32(Pt 2):408–416. 10.1107/S1600577524011706 PMC11892901 39902895

[CR30] Hibbard JVK, Vazquez N, Satija R, Wallingford JB (2021) Protein turnover dynamics suggest a diffusion-to-capture mechanism for peri-basal body recruitment and retention of intraflagellar transport proteins. Mol Biol Cell 32(12):1171–1180. 10.1091/mbc.E20-11-0717 PMC8351562 33826363

[CR31] Hines KE, Middendorf TR, Aldrich RW (2014) Determination of parameter identifiability in nonlinear biophysical models: a Bayesian approach. J Gen Physiol 143(3):401–416. 10.1085/jgp.201311116 PMC3933937 24516188

[CR32] Hoefling M, Lima N, Haenni D, Seidel CAM, Schuler B, Grubmüller H (2011) Structural heterogeneity and quantitative FRET efficiency distributions of polyprolines through a hybrid atomistic simulation and Monte Carlo approach. PLoS ONE 6(5):e19791. 10.1371/journal.pone.0019791 PMC3101224 21629703

[CR33] Hsu DJ, Leshchev D, Kosheleva I, Kohlstedt KL, Chen LX (2021) Unfolding bovine α-lactalbumin with T-jump: characterizing disordered intermediates via time-resolved x-ray solution scattering and molecular dynamics simulations. J Chem Phys 154(10):105101. 10.1063/5.0039194 PMC7943248 33722011

[CR34] Kern N, Frenkel D (2003) Fluid–fluid coexistence in colloidal systems with short-ranged strongly directional attraction. J Chem Phys 118(21):9882–9889. 10.1063/5.0039194. 10.1063/1.1569473

[CR35] Keskin O, Ma B, Rogale K, Gunasekaran K, Nussinov R (2005) Protein-protein interactions: organization, cooperativity and mapping in a bottom-up systems biology approach. Phys Biol 2(2):S24-35. 10.1088/1478-3975/2/2/S03 16204846

[CR36] Kruteva M, Genix AC, Holderer O, Oberdisse J (2022) Recent scattering approaches to structure and dynamics of polymer nanocomposites. Front Soft Matter. 10.3389/frsfm.2022.992563

[CR37] Lee K-H, Dinner AR, Tu C, Campi G, Raychaudhuri S et al (2003) The immunological synapse balances T cell receptor signaling and degradation. Science 302(5648):1218–1222. 10.1126/science.1086507 14512504

[CR38] Liang J, Qian H (2010) Computational cellular dynamics based on the chemical master equation: a challenge for understanding complexity. J Comput Sci Technol 25(1):154–168. 10.1007/s11390-010-9312-6 PMC4079062 24999297

[CR39] Liu JS (2004) Monte Carlo Strategies in Scientific Computing. Springer New York, New York, NY. 10.1007/978-0-387-76371-2

[CR40] Liu R-N, Kang Y-M (2020) Stochastic master equation for early protein aggregation in the transthyretin amyloid disease. Sci Rep 10(1):12437. 10.1038/s41598-020-69319-x PMC7381670 32709875

[CR41] Li D-W, Mohanty S, Irbäck A, Huo S (2008) Formation and growth of oligomers: a Monte Carlo study of an amyloid tau fragment. PLoS Comput Biol 4(12):e1000238. 10.1371/journal.pcbi.1000238 PMC2583953 19057640

[CR42] Loskot P, Atitey K, Mihaylova L (2019) Comprehensive review of models and methods for inferences in bio-chemical reaction networks. Front Genet 10:549. 10.3389/fgene.2019.00549 PMC6588029 31258548

[CR43] McGreevy RL, Pusztai L (1988) Reverse Monte Carlo simulation: a new technique for the determination of disordered structures. Mol Simul 1(6):359–367. 10.1080/08927028808080958

[CR44] McGreevy RL (2001) Reverse Monte Carlo modelling. J Phys-Condens Matter 13(46):R877-913. 10.1088/0953-8984/13/46/201

[CR45] Meng TC, Somani S, Dhar P (2004) Modeling and simulation of biological systems with stochasticity. In Silico Biol 4(3):293–309. 10.3233/ISB-00134 15724281

[CR46] Metropolis N, Rosenbluth AW, Rosenbluth MN, Teller AH, Teller E (1953) Equation of state calculations by fast computing machines. J Chem Phys 21(6):1087. 10.1063/1.1699114 41342507

[CR47] Metropolis N, Ulam S (1949) The Monte Carlo method. J Am Stat Assoc 44(247):335–341. 10.1080/01621459.1949.10483310 18139350

[CR48] Mitra ED, Hlavacek WS (2019) Parameter estimation and uncertainty quantification for systems biology models. Current Opinion in Syst Biol 18:9–18. 10.1016/j.coisb.2019.10.006 PMC7384601 32719822

[CR49] Muñiz‐Chicharro A, Votapka LW, Amaro RE, Wade RC (2023) Brownian dynamics simulations of biomolecular diffusional association processes. Wires Comput Mol Sci. 10.1002/wcms.1649

[CR50] Musino D, Genix A-C, Chaussée T, Guy L, Meissner N et al (2018) Aggregate formation of surface-modified nanoparticles in solvents and polymer nanocomposites. Langmuir 34(9):3010–3020. 10.1021/acs.langmuir.7b03932 29443532

[CR51] Nguyen TD, Chen Y-I, Chen LH, Yeh H-C (2023) Recent advances in single-molecule tracking and imaging techniques. Annu Rev Anal Chem (Palo Alto Calif) 16(1):253–284. 10.1146/annurev-anchem-091922-073057 PMC11729782 37314878

[CR52] Okumura H, Hoai LT, Sompornpisut P, Itoh SG (2026) A lattice Monte Carlo model for amyloid fibril formation. Biophys Physicobiol. 10.2142/biophysico.bppb-v23.0015 PMC13310571 42371580

[CR53] Pappu RV, Cohen SR, Dar F, Farag M, Kar M (2023) Phase transitions of associative biomacromolecules. Chem Rev 123(14):8945–8987. 10.1021/acs.chemrev.2c00814 PMC11513790 36881934

[CR54] Pauw BR, Pedersen JS, Tardif S, Takata M, Iversen BB (2013) Improvements and considerations for size distribution retrieval from small-angle scattering data by Monte Carlo methods. J Appl Crystallogr 46(Pt 2):365–371. 10.1107/S0021889813001295 PMC3627408 23596341

[CR55] Pedersen JS, Møller TL, Corredig M (2026) Scattering from “Babinet” particles (or not…): spherical particles made up of spheres and spherical particles with spherical voids. J Appl Crystallogr 59(Pt 1):59–68. 10.1107/S1600576725010143 PMC12871482 41647188

[CR56] Peter Q, Taylor C, Lapinska U, Wei J, Michaels TCT et al (2026) Oligomers mediate the spatial transmission of Aβ peptide aggregation. Commun Chem 9(1):35. 10.1038/s42004-025-01837-z PMC12823608 41559153

[CR57] Petoukhov MV, Svergun DI (2013) Applications of small-angle X-ray scattering to biomacromolecular solutions. Int J Biochem Cell Biol 45(2):429–437. 10.1016/j.biocel.2012.10.017 23142499

[CR58] Pettigrew MF, Resat H (2007) Multinomial tau-leaping method for stochastic kinetic simulations. J Chem Phys 126(8):084101. 10.1063/1.2432326 17343434

[CR59] Qin S, Zhou H-X (2024) Calculating structure factors of protein solutions by atomistic modeling of protein-protein interactions. Phys A. 10.1016/j.physa.2024.129844 PMC11544587 39525323

[CR60] Qin S, Zhou H-X (2025) Fast calculation of small-angle scattering profiles of dense protein solutions modeled at the all-atom level. Proc Natl Acad Sci U S A 122(39):e2519265122. 10.1073/pnas.2519265122 PMC12501182 40996797

[CR61] Qi SY, Groves JT, Chakraborty AK (2001) Synaptic pattern formation during cellular recognition. Proc Natl Acad Sci U S A 98(12):6548–6553. 10.1073/pnas.111536798 PMC34390 11371622

[CR62] Rana U, Brangwynne CP, Panagiotopoulos AZ (2021) Phase separation vs aggregation behavior for model disordered proteins. J Chem Phys 155(12):125101. 10.1063/5.0060046 34598580

[CR63] Ranganathan S, Shakhnovich EI (2020) Dynamic metastable long-living droplets formed by sticker-spacer proteins. Elife. 10.7554/eLife.56159 PMC7360371 32484438

[CR64] Resat H, Petzold L, Pettigrew MF (2009) Kinetic modeling of biological systems. Methods Mol Biol 541:311–335. 10.1007/978-1-59745-243-4_14 PMC2877599 19381542

[CR65] Rovigatti L, Russo J, Romano F (2018) How to simulate patchy particles⋆. Eur Phys J E Soft Matter 41(5):59. 10.1140/epje/i2018-11667-x 29748868

[CR66] Rubinstein RY, Kroese DP. 2016. Simulation and the Monte Carlo Method. Hoboken, NJ. John Wiley & Sons, Inc. 16(2):307–323. 10.1002/9781118631980

[CR67] Shreider YA (1966) Chapter I: principles of the Monte Carlo method. The Monte Carlo method. Pergamon, Oxford, pp 1–90. 10.1016/C2013-0-01870-1

[CR68] Shemesh A, Ginsburg A, Dharan R, Levi-Kalisman Y, Ringel I, Raviv U (2021) Structure and energetics of GTP- and GDP-tubulin isodesmic self-association. ACS Chem Biol 16(11):2212–2227. 10.1021/acschembio.1c00369 34643366

[CR69] Simon F, Weiss LE, van Teeffelen S (2024) A guide to single-particle tracking. Nat Rev Methods Primers 4(1):66. 10.1038/s43586-024-00341-3

[CR70] Skar-Gislinge N, Camerin F, Stradner A, Zaccarelli E, Schurtenberger P (2023) Using cluster theory to calculate the experimental structure factors of antibody solutions. Mol Pharm 20(5):2738–2753. 10.1021/acs.molpharmaceut.3c00191 PMC10155212 37067466

[CR71] Skar-Gislinge N, Ronti M, Garting T, Rischel C, Schurtenberger P et al (2019) A colloid approach to self-assembling antibodies. Mol Pharm 16(6):2394–2404. 10.1021/acs.molpharmaceut.9b00019 31059276

[CR72] Stradner A, Schurtenberger P (2020) Potential and limits of a colloid approach to protein solutions. Soft Matter. 10.1039/c9sm01953g 31830196

[CR73] Svergun DI, Petoukhov MV, Koch MH (2001) Determination of domain structure of proteins from X-ray solution scattering. Biophys J 80(6):2946–2953. 10.1021/ja069124n PMC1301478 11371467

[CR74] Svergun DI (1999) Restoring low resolution structure of biological macromolecules from solution scattering using simulated annealing. Biophys J 76(6):2879–2886. 10.1016/S0006-3495(01)76260-1 PMC1300260 10354416

[CR75] Székely T, Burrage K (2014) Stochastic simulation in systems biology. Comput Struct Biotechnol J 12(20–21):14–25. 10.1016/j.csbj.2014.10.003 PMC4262058 25505503

[CR76] Tanouye FT, Alves JR, Spinozzi F, Itri R (2023) Unveiling protein-protein interaction potential through Monte Carlo simulation combined with small-angle X-ray scattering. Int J Biol Macromol 248:125869. 10.1016/j.ijbiomac.2023.125869 37473888

[CR77] Taskina A, Magan D, Dannenberg S, Klumpp S (2025) Enhanced condensate fluidity in modified patchy particle models. J Chem Phys. 10.1063/5.0268239 40626470

[CR78] Teixeira PIC, Tavares JM (2017) Phase behaviour of pure and mixed patchy colloids — theory and simulation. Curr Opin Colloid Interface Sci 30:16–24. 10.1063/1.5119359

[CR79] Thompson CJ, Su Z, Vu VH, Wu Y, Leckband DE, Schwartz DK (2020) Cadherin clusters stabilized by a combination of specific and nonspecific cis-interactions. Elife. 10.7554/eLife.59035 PMC7505656 32876051

[CR80] Tiwari NS, van der Schoot P (2016) Stochastic lag time in nucleated linear self-assembly. J Chem Phys 144(23):235101. 10.1063/1.4953850 27334194

[CR81] Tria G, Mertens HDT, Kachala M, Svergun DI (2015) Advanced ensemble modelling of flexible macromolecules using X-ray solution scattering. IUCrJ 2(Pt 2):207–217. 10.1107/S205225251500202X PMC4392415 25866658

[CR82] Ullah M, Wolkenhauer O (2010) Stochastic approaches in systems biology. Wiley Interdiscip Rev Syst Biol Med 2(4):385–397. 10.1002/wsbm.78 20836037

[CR83] Vestergaard CL, Génois M (2015) Temporal Gillespie algorithm: fast simulation of contagion processes on time-varying networks. PLoS Comput Biol 11(10):e1004579. 10.1371/journal.pcbi.1007190 PMC4627738 26517860

[CR84] Vlcek L, Vasudevan RK, Jesse S, Kalinin SV (2017) Consistent integration of experimental and ab initio data into effective physical models. J Chem Theory Comput 13(11):5179–5194. 10.1021/acs.jctc.7b00114 28892618

[CR85] Von Neumann J (1963) Various techniques used in connection with random digits. In: Taub AH (ed) John von Neumann: collected works, Vol. 5. Pergamon Press, Oxford, pp 768–770

[CR86] Weikl TR, Lipowsky R (2004) Pattern formation during T-cell adhesion. Biophys J 87(6):3665–3678. 10.1529/biophysj.104.045609 PMC1304881 15377531

[CR87] Weimann L, Ganzinger KA, McColl J, Irvine KL, Davis SJ et al (2013) A quantitative comparison of single-dye tracking analysis tools using Monte Carlo simulations. PLoS ONE 8(5):e64287. 10.1371/journal.pone.0064287 PMC3667770 23737978

[CR88] Winterer M, Geiß J (2023) Combining reverse Monte Carlo analysis of X-ray scattering and extended X-ray absorption fine structure spectra of very small nanoparticles. J Appl Crystallogr 56(Pt 1):103–109. 10.1107/S1600576722010858 PMC9901930 36777145

[CR89] Young WM, Elcock EW (1966) Monte Carlo studies of vacancy migration in binary ordered alloys: I. Proc Phys Soc 89(3):735–746. 10.1088/0370-1328/89/3/329

[CR90] Zaccarelli E, Sciortino F, Tartaglia P (2007) A spherical model with directional interactions. I Static properties. J Chem Phys 127(17):174501. 10.1063/1.2799522 17994821

[CR91] Zhao C, Shukla D (2018) SAXS-guided enhanced unbiased sampling for structure determination of proteins and complexes. Sci Rep 8(1):17748. 10.1038/s41598-018-36090-z PMC6288155 30531946

[CR92] Zhou M, Wang R (2026) Exploring protein–protein interactions: concepts, methods, and implications. In: Zhang J (ed) Targeting protein–protein interactions for drug discovery. Wiley, pp 1–28. 10.1002/9783527845828.ch1

